# Multi-Spectral Band Analysis for Satellite-to-Aerial Image Registration: A Comparative Study of Deep Learning and Traditional Feature-Matching Methods

**DOI:** 10.3390/s26134165

**Published:** 2026-07-02

**Authors:** Dongyeob Han, Jeong Heon Song, Sun-Gu Lee

**Affiliations:** 1 Department of Civil Engineering, Chonnam National University, Gwangju 61186, Republic of Korea; hozilla@chonnam.ac.kr; 2 National Satellite Operation & Application Center, Korea Aerospace Research Institute, Daejeon 34133, Republic of Korea; leesg@kari.re.kr

**Keywords:** image registration, multi-spectral analysis, LightGlue, LoFTR, RoMa, SIFT, KOMPSAT-3A, HD map, feature matching, CLAHE

## Abstract

**Highlights:**

**What are the main findings?**

- Across seven spectral bands and seven patch sizes, panchromatic-derived composites (EMPPAN, BT601) and the red band provide the most stable matching, while the detector-free deep matchers added herein (LoFTR, RoMa) yield the densest, most stable correspondences and the lowest internal registration RMSE.
- When evaluated against independent network GNSS check points, all matchers—traditional and deep—reach a similar registration accuracy of approximately 2.8 m, demonstrating that matcher choice influences correspondence density and transformation stability more than independent geodetic accuracy.

**What are the implications of the main findings?**

- For the tested KOMPSAT-3A/VWorld setting, matcher selection is primarily a throughput and robustness decision; the achieved meter-level accuracy is suitable for HD map preprocessing and candidate GCP generation rather than final lane-level map production.
- Edge-based FFT methods are unsuitable for cross-resolution satellite-to-aerial registration, and CLAHE preprocessing must be applied selectively based on the matching algorithm.

**Abstract:**

Precise geometric registration between high-resolution satellite imagery and aerial orthophotos is essential for generating high-definition (HD) maps that support autonomous vehicle navigation. This study presents a comprehensive evaluation of multi-spectral band performance for image registration between KOMPSAT-3A satellite imagery (0.55 m resolution) and VWorld aerial orthophotos (0.25 m resolution) across seven patch size configurations. Five feature-matching approaches were systematically compared: LightGlue with CLAHE preprocessing, edge-based FFT methods (with and without CLAHE), and SIFT-based methods (with and without CLAHE). Two additional detector-free deep matchers, LoFTR and RoMa, were further integrated into the same pipeline for comparison. The experimental results reveal significant variations in registration accuracy across spectral bands, with the panchromatic-derived products (SPECPAN and EMPPAN) and luminance composite BT601 image demonstrating superior stability compared to individual visible and NIR bands. LightGlue achieved consistently high inlier counts (averaging 1100+ matched points) across all spectral bands and patch configurations, while SIFT with CLAHE preprocessing yielded the lowest matching RMSE (averaging 1.55 pixels). Among all matchers, the detector-free methods produced the densest and most stable correspondences, with LoFTR giving the best transformation stability, whereas edge-based methods were markedly less stable. However, an independent assessment against network GNSS check points showed a registration accuracy of approximately 2.8 m that was statistically similar across all matchers, indicating that matcher selection mainly affects correspondence density and transformation stability rather than independent geodetic accuracy. The achieved meter-level accuracy is suitable for HD map preprocessing and candidate GCP generation rather than final lane-level mapping, and the reported guidance is specific to the tested KOMPSAT-3A/VWorld setting.

## 1. Introduction

High-definition (HD) maps have emerged as a critical enabling technology for autonomous vehicle navigation, providing centimeter-level accurate representations of road geometry, lane markings, traffic infrastructure, and other navigational elements essential for safe self-driving operations [1,2]. Unlike conventional navigation maps designed for human drivers, HD maps require horizontal positional accuracies of 10–20 cm and vertical accuracies of 20–30 cm to support lane-level localization and path planning algorithms [1]. The generation and maintenance of such precise spatial databases at national or global scales present significant technical and economic challenges, motivating research into scalable production methods that leverage remote sensing data sources.

HD map generation from satellite and aerial imagery has become an active research area in response to advances in autonomous driving technologies [1,2]. Liu et al. [1] reviewed HD map requirements, identifying a horizontal accuracy of 10–20 cm as essential for lane-level navigation. Jeong et al. [3] provided comprehensive tutorials on HD map generation for urban environments, discussing integration of multi-source remote sensing data. Bao et al. [2] surveyed HD map creation methods, noting that satellite imagery at 50 cm/pixel or better enables scalable road extraction. Recent studies have further emphasized that HD maps must be continuously generated, verified, and updated to support practical autonomous driving deployment. Asrat and Cho [4] reviewed recent progress in HD map generation and maintenance, highlighting that map updating remains a major challenge for scalable autonomous driving systems. Kwag and Toth [5] reviewed end-to-end HD map generation approaches and discussed the importance of integrating multiple sensing modalities and map representations.

Road extraction algorithms leveraging deep learning have achieved significant progress. Zhou et al. [6] developed D-LinkNet for high-resolution satellite road extraction, winning the DeepGlobe Road Extraction Challenge. Wei et al. [7] proposed weakly supervised approaches for large-scale road extraction from high-resolution imagery, while Ma et al. [8] introduced BoundaryNet, combining mobile laser scanning and satellite data for road boundary extraction. Chen et al. [9] recently surveyed deep learning road extraction methods, identifying precise geometric registration as a critical preprocessing requirement.

Satellite imagery offers compelling advantages for HD map production, including wide-area coverage, regular revisit capabilities, and decreasing costs of sub-meter resolution data [10,11]. Contemporary very-high-resolution (VHR) satellites such as KOMPSAT-3A, WorldView-3, and Pleiades provide panchromatic ground sampling distances (GSDs) of 0.3–0.55 m, approaching the spatial resolution requirements for extracting road-level features [12,13]. However, achieving the positional accuracy demanded by HD map applications requires precise geometric registration of satellite imagery to higher-accuracy reference datasets, typically aerial orthophotos with 10–25 cm GSDs that have been rigorously orthorectified using dense ground control networks. Satellite vendors generally provide rational polynomial coefficients (RPCs) for initial geometric sensor modeling. However, to elevate the positional accuracy to the level required for high-precision topographic mapping, these RPCs require rigorous bias compensation via accurate ground control points (GCPs).

The registration of multi-spectral satellite imagery to aerial orthophotos presents several technical challenges arising from differences in spectral response, spatial resolution, illumination geometry, and temporal acquisition conditions [14,15]. Feature-based registration methods, which extract and match distinctive image features between source and reference datasets, have demonstrated robust performance for such multi-modal registration tasks [16,17]. Traditional approaches based on the Scale-Invariant Feature Transform (SIFT) and related descriptors have been widely employed for remote sensing image registration, achieving sub-pixel accuracy under favorable conditions [18,19]. More recently, deep learning-based feature matchers have substantially reshaped this landscape along two complementary directions. The detector–descriptor–matcher pipeline has been advanced by SuperPoint, SuperGlue, and LightGlue [20,21,22], which deliver high inlier density and run-time efficiency on multi-temporal imagery. In parallel, detector-free transformer-based matching introduced by LoFTR [23] removed the dependence on a separate keypoint stage and demonstrated robust dense correspondence in low-texture regions, while the most recent dense robust matching approaches, such as RoMa [24], further extend this line by handling extreme appearance variation.

A critical yet underexplored aspect of satellite-to-aerial registration is the selection of optimal spectral bands for feature matching. Multi-spectral satellites typically acquire imagery in blue, green, red, and near-infrared (NIR) bands alongside panchromatic channels, each exhibiting different characteristics for feature detection and matching [25,26]. The spectral response differences between satellite and aerial sensors, combined with band-specific noise characteristics and atmospheric effects, can significantly impact registration performance [27]. Furthermore, pan-sharpened products combining multi-spectral and panchromatic data offer potential advantages through enhanced spatial detail while preserving spectral information [28].

Although many image registration and feature-matching studies have been reported, practical guidance remains limited for satellite-to-aerial registration workflows used in HD map preprocessing. In such workflows, the objective is not only to obtain visually good image alignment but also to generate a sufficient number of spatially reliable GCPs for geometric correction of high-resolution satellite imagery. The performance is strongly affected by the spectral band used for matching, the spatial resolution difference between satellite and aerial imagery, preprocessing, patch size, and the robustness of the matching algorithm.

In this study, therefore, we focus on an engineering-oriented evaluation of band selection and matching method stability for KOMPSAT-3A imagery registered to VWorld aerial orthophotos. We systematically compare seven spectral band representations—including the panchromatic-derived composites SPECPAN and EMPPAN introduced in this work—five matching methods, and seven patch size configurations, and evaluate each combination under both within-footprint accuracy (affine RMSE-Ref) and extrapolation stability beyond the reference region (affine RMSE-DEM). The output is a decision table indicating which (band, method, patch size) combinations are sufficient for automated GCP generation in HD map preprocessing pipelines, and which are not.

The primary contributions of this work include (1) quantitative comparison of registration accuracy across multiple spectral bands, identifying optimal band selection strategies; (2) evaluation of deep learning versus traditional feature-matching methods for cross-resolution satellite-to-aerial registration; (3) analysis of patch size effects on registration stability and accuracy; and (4) practical recommendations for satellite-based HD map preprocessing workflows requiring robust sub-meter positional accuracy.

## 2. Matching Methods

### 2.1. Multi-Spectral Image Registration

Multi-spectral image registration has been extensively studied in the remote sensing literature, with research demonstrating significant performance variations across spectral bands. Yi et al. [25] proposed orientation-restricted SIFT for multi-spectral satellite image registration, finding that restricting descriptor orientation improved matching between bands with different spectral responses. Teke et al. [26] conducted comprehensive evaluations of SIFT and SURF for high-resolution multi-spectral matching, reporting that edge-based descriptors outperformed intensity-based approaches for cross-band registration. Aguilera et al. [29] developed specialized multi-spectral interest point detectors optimized for visible–NIR image pairs, achieving improved repeatability compared to standard detectors.

The challenge of NIR–visible registration has received particular attention due to the nonlinear intensity differences between these spectral regions [29]. Firmenichy et al. [30] demonstrated that edge-based features provide more reliable correspondences than intensity-based descriptors for RGB-NIR matching. Saleem and Sablatnig [31] proposed modified SIFT descriptors incorporating gradient orientation normalization to improve matching under spectral variations. More recent work by Zhao and Zhang [32] introduced heterogeneous self-supervised interest point matching specifically designed for multi-modal remote sensing registration.

### 2.2. Traditional Registration and Matching Methods

SIFT remains a foundational algorithm for remote sensing image registration, with numerous variants developed to address specific challenges [18]. Ma et al. [19] proposed a modified SIFT with enhanced feature matching, achieving improved registration accuracy for remote sensing applications. Sedaghat et al. [33] introduced adaptive binning SIFT descriptors for satellite imagery, reporting RMSE values of approximately 0.44 pixels on multi-temporal optical images. Meanwhile, OS-SIFT [34] addressed optical-to-SAR registration through robust SIFT-like descriptors designed for heterogeneous image pairs.

Frequency domain methods based on phase correlation provide alternative approaches with inherent robustness to noise and illumination differences [35]. Reddy and Chatterji [36] demonstrated FFT-based registration, achieving translation, rotation, and scale-invariant matching. Foroosh et al. [37] extended phase correlation to sub-pixel registration accuracy, while Wu et al. [38] proposed a multiple polar Fourier transform for similarity transform estimation, achieving 1/10- to 1/100-pixel accuracy under favorable conditions.

### 2.3. Deep Learning Feature Matching

Deep learning approaches have transformed feature matching in computer vision, with architectures such as SuperPoint [20], SuperGlue [21], and LightGlue [22] demonstrating state-of-the-art performance on challenging matching benchmarks. SuperPoint introduced self-supervised training for joint interest point detection and description, learning features robust to viewpoint and illumination changes. SuperGlue extended this with graph neural network-based matching, incorporating geometric reasoning, and LightGlue further improved efficiency while maintaining accuracy through adaptive computation mechanisms. In parallel, detector-free transformer-based matching introduced by LoFTR [23] established an alternative family that bypasses the explicit keypoint stage and is widely regarded alongside LightGlue as a current state-of-the-art baseline. In the present study, we evaluate LightGlue because its detector–descriptor–matcher pipeline can be integrated more cleanly with the patch-based GCP extraction workflow used for RPC refinement, while LoFTR remains a natural candidate for a future detector-free comparison.

Application of these methods to remote sensing imagery has shown promising results. Recent evaluations on satellite stereo pairs demonstrated that LightGlue and SuperGlue significantly outperform traditional methods on multi-temporal imagery with seasonal and illumination variations [39]. Studies applying SuperGlue to large aerial image matching achieved improved performance in weak texture survey areas, including mountains and grasslands [40]. Research on UAV-to-satellite cross-view matching using LightGlue reported robust performance despite significant viewpoint differences [41].

## 3. Materials and Methods

### 3.1. Study Data

**Figure 1:**
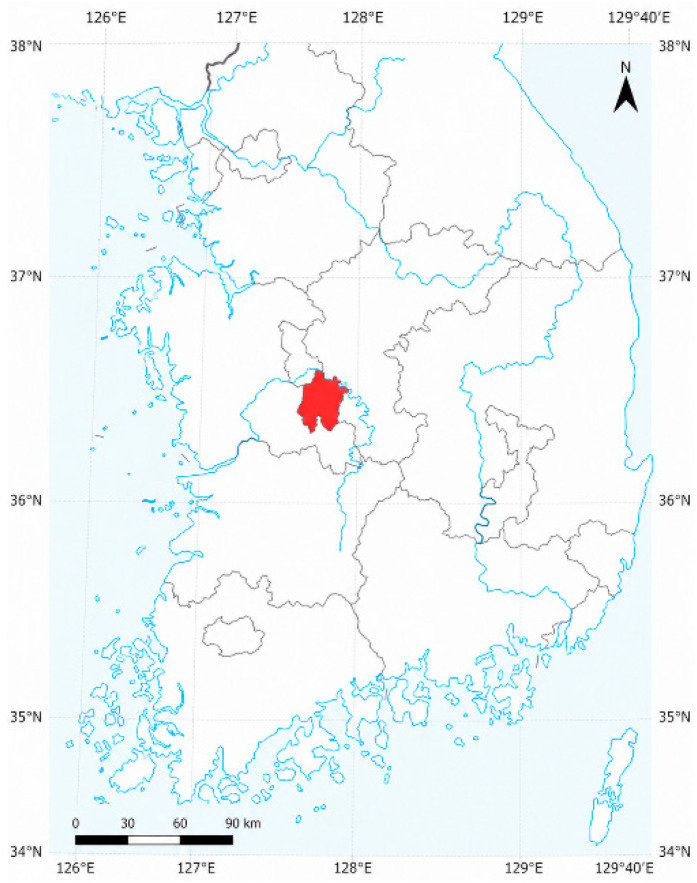
Geographic location of the study area in South Korea, with Daejeon shown in red.

**Figure 2:**
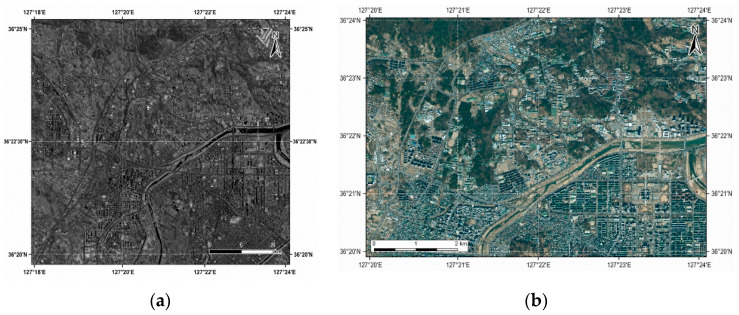
Example KOMPSAT-3A image and corresponding VWorld aerial orthophoto used for registration: (**a**) KOMPSAT-3A NIR band; (**b**) VWorld.

The experiments used Daejeon’s KOMPSAT-3A (Korean Multi-Purpose Satellite 3A; Korea Aerospace Research Institute, Daejeon, Republic of Korea) satellite imagery as the source dataset requiring registration (Figure 1 and Figure 2a). KOMPSAT-3A provides high-resolution imagery with 0.55 m panchromatic and 2.2 m multi-spectral GSD from a 528 km sun-synchronous orbit [12]. Seo et al. [12] conducted geometric calibration and validation of the AEISS-A camera system, establishing baseline accuracy characteristics, while Jin et al. [13] performed radiometric calibration with uncertainty analysis, achieving 4.27% radiometric uncertainty. Yeom et al. [42] developed methods for updating absolute radiometric characteristics using pseudo-invariant calibration sites, and recent work on multi-strip image mosaicking achieved average registration errors of 1.63 pixels using homography transformation on KOMPSAT-3A scenes [43].

The ground-truth reference dataset consisted of VWorld aerial orthophotos with approximately 0.25 m GSD (Figure 2b). VWorld represents the national spatial data infrastructure of South Korea, providing highly accurate spatial information datasets. This resolution ratio of approximately 2.2:1 between source and reference imagery presents a realistic scenario for HD map production workflows where satellite data must be registered to higher-resolution aerial reference frames.

The satellite imagery and aerial orthophotos used in this study were not acquired simultaneously. The KOMPSAT-3A scene was acquired on 28 March 2022 at approximately 04:44 UTC, with a sun elevation of about 55° and a sun azimuth of about 214°. The VWorld aerial orthophoto used as the reference dataset was from 2023; therefore, the two datasets were not same-year observations. However, the 2023 VWorld orthophoto was selected because it was seasonally similar to the spring acquisition period of the KOMPSAT-3A image, which helps reduce major seasonal differences in vegetation and surface appearance compared with references from other seasons. Detailed tile-level acquisition metadata for the VWorld orthophoto, including exact imaging time, sun angle, cloud cover, and weather conditions, were not available. Therefore, the experiment includes unavoidable temporal and radiometric differences between the satellite and aerial datasets. These differences may influence feature detectability through changes in shadows, illumination, vegetation condition, road surface appearance, and land cover changes.

Seven spectral band configurations were evaluated for registration performance: blue (B), green (G), red (R), near-infrared (NIR), SPECPAN (synthetic spectral overlap panchromatic simulation), BT601 (RGB luma grayscale composite), and EMPPAN (empirical RGB pseudo-panchromatic composite).

- BT601 (ITU-R BT.601 luminance): Calculated as (I = 0.114 × B + 0.587 × G + 0.299 × R), this standard luminance formula prioritizes green wavelengths for balanced visible contrast, commonly used in video processing [44].
- EMPPAN: Defined as PAN = 0.1×B + 0.3×G + 0.6×R, this RGB-only grayscale composite was introduced as an empirical pseudo-panchromatic variant to emphasize visible-band contrast, particularly in the red wavelengths where road surface features exhibit strong gradients. The EMPPAN weights were heuristically selected to emphasize visible-band contrast, particularly in the red channel, where road surfaces and lane-related structures showed strong gradients in the study area. These weights were not optimized through a training procedure. The Gaussian low-pass filter with σ = 0.6 was applied selectively only to the filtered EMPPAN variant to evaluate whether slight smoothing improved matching robustness; it was not applied to all spectral band experiments.
- A controlled ablation was performed to verify that the performance of EMPPAN does not depend on the particular heuristic weights, and to separate the effect of the spectral weighting from the optional Gaussian smoothing. Co-located KOMPSAT-3A and VWorld patches were extracted at matched control point locations (fixed geometry) and matched with SuperPoint + LightGlue under eight RGB weightings (pure blue, green, and red, equal weights, BT601, EMPPAN, and green- and red-emphasized variants), each evaluated with and without the σ = 0.6 Gaussian low-pass filter applied uniformly to both images. The inlier count and matching RMSE were averaged over 60 spatially distributed patches with 95% bootstrap confidence intervals.
- SPECPAN: PAN = 0.20×B + 0.23×G + 0.17×R + 0.40×NIR. These weights were computed by the authors from the nominal overlap between the KOMPSAT-3A panchromatic band (450–900 nm) and the multi-spectral bands, and they were then normalized to sum to one [12,45].

To provide an external accuracy assessment that is independent of the feature-matching pipeline, nine ground points were surveyed in the study area using network RTK-GNSS with an approximate three-dimensional precision of 0.10 m. Six of these points fall within the KOMPSAT-3A and VWorld data. Because the automatic registration relies exclusively on image features matched between the KOMPSAT-3A and VWorld data, these surveyed points were never used in the estimation of any transformation and, therefore, act as fully independent check points (CPs). For each registration configuration, the estimated image space affine was applied to the CP image positions, and the resulting ground coordinates were compared with the surveyed coordinates to obtain an independent planimetric RMSE (RMSE-CP).

### 3.2. Feature-Matching Methods

The following five feature-matching approaches were systematically evaluated.

- LightGlue with CLAHE: The LightGlue architecture combines SuperPoint feature detection with efficient attention-based matching. Contrast-limited adaptive histogram equalization (CLAHE) preprocessing was applied to enhance local contrast and improve feature detection in areas with low radiometric variation on KOMPSAT-3A imagery.
- EdgeFFT: Edge-based feature extraction followed by FFT-based phase correlation matching leverages gradient information that tends to be more consistent across spectral bands than raw intensity values.
- EdgeFFT with CLAHE: Using the EdgeFFT method with CLAHE preprocessing enhances edge detection in low-contrast regions prior to frequency domain correlation.
- SIFT: Standard Scale-Invariant Feature Transform implementation provides baseline performance for traditional localized feature matching.
- SIFT with CLAHE: SIFT feature extraction can be paired with CLAHE preprocessing to improve keypoint detection across varying illumination conditions.

LightGlue was evaluated with CLAHE preprocessing because preliminary tests without CLAHE produced insufficiently reliable correspondences for several spectral bands and patch size configurations. After RANSAC-based geometric verification, the remaining inliers were often too few or too unevenly distributed to solve a stable affine transformation. Consequently, valid registration metrics such as RMSE After transformation and affine RMSE could not be consistently computed for LightGlue without CLAHE. Therefore, only the LightGlue + CLAHE configuration was included in the final quantitative comparison. SuperPoint was used as the local feature extractor with a maximum of 2048 keypoints and a detection threshold of 0.0005. LightGlue was used with SuperPoint features, 9 layers, depth confidence of 0.95, width confidence of 0.99, and a minimum matching score of 0.02. For patch-level geometric verification, LightGlue matches were filtered using RANSAC-based affine partial transformation estimation with a reprojection threshold of 1.2 pixels, confidence of 0.999, and 3000 maximum iterations.

### 3.3. Patch Size Configuration

Registration was evaluated across seven patch size configurations to assess the relationship between the processing window size and matching performance (Table 1). The patch sizes were defined separately for HRSI (high-resolution satellite imagery) and VWorld reference data to account for resolution differences:

The seven patch size configurations were selected to evaluate matching performance across different spatial contexts while maintaining comparable ground coverage between KOMPSAT-3A and VWorld image windows. Because the KOMPSAT-3A GSD is approximately 0.55 m and the VWorld GSD is approximately 0.25 m, the VWorld patch sizes were set to maintain an approximate 2.2:1 resolution ratio relative to the HRSI patches. The configurations span fine-scale matching windows (131 × 131 HRSI pixels; approximately 72 m ground coverage), which are suitable for dense local correspondence, to coarse-scale windows (651 × 651 HRSI pixels; approximately 358 m ground coverage), which provide broader road segment and road network context for robust initial alignment. The intervals between configurations are non-uniform because they were chosen to represent practical matching scales rather than equal pixel increments, allowing for evaluation of the tradeoff between local feature detail, contextual robustness, and computational cost.

### 3.4. Evaluation Metrics

Registration performance was assessed using multiple complementary metrics:

- Total GCPs: The total number of ground control point correspondences identified by each matching method, indicating the density of the correspondence field.
- Inliers: The number of correspondences passing RANSAC-based geometric verification, representing reliable matches consistent with the estimated transformation model.
- Inlier Rate: The ratio of inliers to total GCPs expressed as a percentage, indicating matching reliability and the proportion of correct correspondences.
- RMSE Before: Root Mean Square Error of matched point positions before geometric transformation, representing initial misregistration magnitude.
- RMSE After: RMSE After applying the estimated affine transformation to matched GCPs, indicating transformation fitting accuracy.
- Improvement: Percentage reduction in RMSE from before to after transformation, quantifying registration effectiveness.
- Affine RMSE-Ref: RMSE evaluated on sampled grid GCPs over the VWorld reference extent, assessing transformation accuracy within the calibration region.
- Affine RMSE-DEM: RMSE of the image space affine approximation evaluated over an extended grid covering the DEM extent. This metric does not represent validation against independent DEM control points. Instead, the DEM provides terrain height values used in RPC projection for sampled grid locations. A 20 × 20 grid was sampled over the DEM-covered extent, the RPC bias model was evaluated at these grid locations, and a 2D affine approximation was fitted in image space. The resulting RMSE, expressed in image pixels, indicates the stability of the affine approximation when extrapolated beyond the VWorld reference image extent.

## 4. Results

### 4.1. Feature-Matching Performance

Table 2 presents the feature-matching results for the baseline patch configuration (HRSI: 131, VWorld: 289). LightGlue with CLAHE consistently produced the highest number of total correspondences across all spectral bands, ranging from 2001 (blue) to 2175 (red) GCPs. For patch configuration 1, after RANSAC filtering, LightGlue + CLAHE maintained the highest inlier counts across spectral bands, with 702–1046 verified correspondences. The maximum value of 1046 inliers was obtained for the EMPPAN band, indicating that EMPPAN provided particularly high correspondence density in this configuration. This range should be interpreted as the band-wise result for configuration 1.

The SIFT-based methods produced moderate GCP counts ranging from 106 (NIR without CLAHE) to 495 (EMPPAN with CLAHE). CLAHE preprocessing substantially improved SIFT performance, particularly for the NIR band, where inlier counts increased from 54 to 137 (refer to Table 2). The EdgeFFT methods generated fewer correspondences overall, with notably reduced effectiveness when CLAHE was applied, suggesting that histogram equalization may interfere with the edge-based correlation process.

Inlier rates, which indicate each algorithm’s ability to reject false matches (e.g., confusing similar-looking roof tiles or road segments), varied substantially across methods. SIFT without CLAHE achieved the highest inlier rates (24.8–50.9%), indicating that while fewer correspondences were detected, a higher proportion were geometrically consistent. LightGlue inlier rates ranged from 35.1% to 48.4%, representing a favorable balance between massive correspondence density and matching reliability. EdgeFFT methods showed variable inlier rates from 20.8% to 38.9%, with performance dependent on spectral band characteristics.

### 4.2. Registration Accuracy Analysis

Initial misregistration (RMSE Before) was consistent across methods, ranging from 15.79 to 17.95 pixels, confirming comparable initial conditions prior to alignment. After affine transformation fitting, RMSE After values demonstrated substantial variation in localized registration accuracy. SIFT with CLAHE achieved the lowest post-registration RMSE across most bands, with values ranging from 1.29 pixels (red) to 1.72 pixels (EMPPAN), and LightGlue produced slightly higher but consistent RMSE After values of 2.13–2.51 pixels across all bands.

The improvement percentages quantify registration effectiveness relative to initial misalignment. SIFT with CLAHE achieved the greatest improvements, reaching 91.9% for the red band, while LightGlue improvements ranged from 84.2% to 86.7% and EdgeFFT methods showed comparable improvements of 86.9–90.8% where successful. These results indicate that all methods achieved substantial geometric correction when sufficient correspondences were available.

To address the absence of repeated trials in the deterministic pipeline, the variability of the registration accuracy was quantified with a spatial bootstrap. The scene was partitioned into a 6 × 6 grid of tiles and, for each configuration, the per-point registration residuals were resampled at the tile level (cluster bootstrap, 4000 replicates) to obtain 95% confidence intervals of the RMSE. Differences between spectral bands were assessed with a spatially paired bootstrap over the tiles shared by each band pair; a difference was regarded as statistically significant when its 95% confidence interval excluded zero.

### 4.3. Spectral Band Performance Comparison

Analysis across spectral bands revealed distinct performance patterns related to band characteristics. The visible bands (B, G, R) showed consistent feature matching with all methods, benefiting from strong correspondence with the aerial reference imagery acquired in similar spectral ranges. The NIR band presented greater challenges, particularly for methods without CLAHE preprocessing, due to spectral response differences between satellite and aerial sensors.

The panchromatic-derived and composite bands showed different strengths depending on the evaluation metric. For the specific LightGlue + CLAHE result in configuration 1, EMPPAN produced 1046 inliers with a 48.4% inlier rate, indicating strong correspondence density. However, the method-averaged results in Table 3 show that EMPPAN had an average RMSE After of 1.96 px, which was higher than the red band (1.69 px), SPECPAN (1.84 px), and BT601 (1.84 px). Therefore, EMPPAN should be interpreted as advantageous for generating dense correspondences rather than as the most accurate band in terms of local registration error. The red band provided the lowest average RMSE After, while SPECPAN and BT601 offered balanced performance between correspondence density and registration accuracy.

The values in Table 3 were computed by averaging each metric across the evaluated matching methods with equal weighting. Failed or unavailable cases were excluded from the corresponding average. Thus, Table 3 summarizes method-averaged band behavior rather than the performance of a single matching algorithm.

### 4.4. Patch Size Effects

Registration performance exhibited complex relationships with patch size configuration. Configurations 1, 4, and 7 were selected as representative small-, medium-, and large-patch cases for discussion, while complete results for all seven configurations are provided in the Supplementary Materials. Table 4 and Table 5 detail the breakdown for mid- and maximum-size patches, and Table 6 summarizes results across all seven patch configurations. For LightGlue, larger patches generally increased correspondence counts and improved RMSE After up to medium-to-large configurations, after which the gains saturated or partially reversed. As shown in Table 6, LightGlue with CLAHE produced average inlier counts ranging from 896 to 1385 across the seven patch size configurations. The highest average inlier count was observed for configuration 5, while the lowest was observed in configuration 1, confirming that LightGlue provided the densest correspondence field among the evaluated methods, though the exact number of inliers varied with patch size and spectral band.

SIFT-based methods showed more pronounced patch size sensitivity. Larger patches enabled the detection of more features, with SIFT + CLAHE generating up to 2444 total GCPs at configuration 7 (HRSI: 651, VWorld: 1433). However, inlier rates decreased with larger patches, suggesting an increased susceptibility to mismatches over extended spatial extents.

EdgeFFT methods exhibited degraded performance with larger patches, producing insufficient correspondences for reliable transformation estimation at configurations 6 and 7. This limitation reflects the localized nature of edge-based correlation, which becomes less effective as patch sizes exceed optimal ranges for the underlying image structure. From a frequency domain perspective, EdgeFFT estimates displacement from correlation peaks derived from edge or gradient structures. As the patch size increases, the window includes more heterogeneous land cover content, repeated road and building patterns, shadows, and low-frequency background variation. These factors can introduce multiple competing frequency components or broaden the dominant correlation peak, reducing the stability of the estimated displacement. Therefore, larger patches do not necessarily improve EdgeFFT performance, even though they provide broader spatial context.

### 4.5. Transformation Stability Assessment

The affine RMSE-Ref and RMSE-DEM metrics provide critical insight into transformation stability, which is particularly relevant for mapping applications requiring accurate extrapolation of the geometric model beyond densely matched regions. Table 7 presents stability analysis results for the baseline configuration.

LightGlue demonstrated the most consistent stability with affine RMSE-Ref values ranging from 0.30 to 1.76 pixels and RMSE-DEM values of 1.21–7.10 pixels. The relatively low variance across spectral bands indicates robust transformation estimation benefiting from the high correspondence density.

SIFT with CLAHE achieved comparable stability with RMSE-Ref values of 0.16–1.77 pixels, though RMSE-DEM showed greater variability (0.66–7.18 pixels). The NIR band exhibited elevated RMSE-DEM (4.60 pixels) compared to visible bands, reflecting increased extrapolation uncertainty.

EdgeFFT methods showed substantially higher and more variable stability metrics. RMSE-DEM values ranged from 0.72 to 9.98 pixels for EdgeFFT, with EdgeFFT + CLAHE producing particularly unstable results (up to 16.33 pixels for the green band). These findings indicate that while edge-based methods may achieve occasional local accuracy on small patches, the sparse correspondence distribution compromises transformation stability over extended spatial coverage.

## 5. Discussion

### 5.1. Optimal Spectral Band Selection

The experimental results provide clear guidance for spectral band selection in satellite-to-aerial registration workflows. The panchromatic-derived bands, particularly EMPPAN and BT601, demonstrated consistently superior performance across multiple metrics, an advantage stemming from several factors: (1) the broad spectral coverage of panchromatic bands reduces spectral mismatch with visible band aerial references; (2) enhanced spatial detail from pan-simulation and empirical RGB pseudo-compensation improves feature localization; and (3) higher signal-to-noise ratios in aggregated spectral bands support more reliable feature detection.

Among individual spectral bands, the green band showed favorable characteristics with balanced performance across all methods. The red band achieved the lowest RMSE After values with SIFT methods, suggesting an optimal gradient structure for scale-space feature detection. The NIR band presented the greatest challenges, particularly without preprocessing, consistent with the literature findings on visible–NIR registration difficulties [29,30]. For operational HD map production, we recommend EMPPAN or BT601 as primary registration bands, with the green band serving as a fallback for multi-spectral-only acquisitions.

### 5.2. Method Selection Considerations

The choice between LightGlue and SIFT-based methods involves tradeoffs between correspondence density, local precision, and global stability. LightGlue provides consistently high correspondence density, with average inlier counts of 896–1385 across patch size configurations, and stable transformation estimation, making it suitable for automated processing pipelines where robust performance across varying conditions is prioritized. The dense correspondence distribution supports reliable affine parameter estimation and enables quality assessment through residual analysis.

SIFT with CLAHE preprocessing achieved the lowest RMSE After values, which is beneficial for applications requiring maximum local geometric accuracy. However, the lower correspondence density increases sensitivity to outlier contamination and may compromise transformation stability in areas with sparse features. The method remains valuable for high-precision registration of limited extents where dense correspondences are not required.

EdgeFFT methods proved unsuitable for this cross-resolution registration task, exhibiting degraded performance with larger patches and unstable transformation extrapolation. The frequency domain correlation approach appears better suited to same- or similar-resolution image pairs where phase relationships are more consistent [36,37].

### 5.3. Implications for HD Map Production

The achieved registration accuracies must be interpreted in the context of precision requirements. With RMSE After values of 1.3–2.5 pixels at 0.55 m GSD, the geometric accuracy translates to approximately 0.7–1.4 m in ground coordinates. While this exceeds the 10–20 cm accuracy requirement for lane-level positioning [1], several considerations apply.

**Figure 3:**
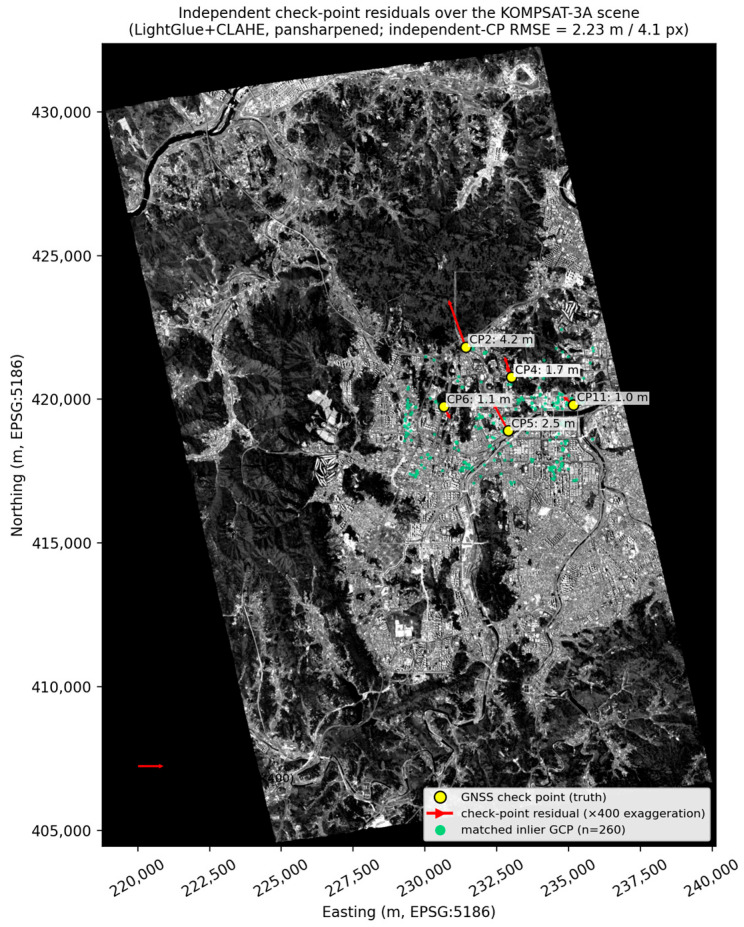
Independent check point residual vector map over the KOMPSAT-3A scene for the representative pansharpened LightGlue + CLAHE configuration. Yellow markers denote the six network GNSS check points; red arrows show the planimetric registration error (exaggerated ×400) with per-point magnitudes; green dots show the spatial distribution of matched inlier GCPs. Independent check point RMSE = 2.23 m (4.05 px).

To verify these fitting-based accuracies against external ground truth, the registration results were additionally evaluated using the six independent GNSS check points described in Section 3.1. For the representative pansharpened LightGlue + CLAHE configuration, the independent check point RMSE was 2.23 m (4.05 px), with per-point planimetric errors of 0.86–4.21 m (Figure 3). Across the pansharpened feature-driven configurations, the independent check point RMSE ranged from 1.6 to 3.3 m (2.9–6.0 px). This independent error is approximately 2.3 times the matched-point fitting residual (median across configurations), confirming that the fitting residual alone tends to understate the true geometric accuracy. The independent check point errors fall within the affine RMSE-DEM stability range reported above, indicating that the extrapolation-based stability metric is a realistic proxy for independent geodetic accuracy. The relative ranking of configurations was preserved under independent validation (Pearson r = 0.77 between the fitting residual and the independent check point RMSE), so the comparative conclusions of this study remain valid.

These results confirm that the achieved accuracy is at the meter level (approximately 1.6–3.3 m) rather than the 0.10–0.20 m lane-level accuracy required for final HD map production. The registration is, therefore, suitable for preprocessing and automated candidate GCP generation, whereas final HD map production would require additional rigorous sensor model adjustment against an independent control. We note that the present independent assessment is based on six surveyed points concentrated in part of the scene; it anchors the absolute accuracy rather than providing a spatially exhaustive validation.

First, the affine transformation model employed provides global geometric correction but cannot account for local terrain-induced distortions. Integration with accurate DEM data and rational polynomial coefficient (RPC) refinement would improve local accuracy. Second, the reported RMSE values represent the transformation fitting error on matched points; systematic biases from sensor modeling or the absolute accuracy of the reference dataset contribute additional real-world uncertainty. Finally, HD map features extracted from registered imagery undergo subsequent refinement through vectorization, conflation with existing data, and field verification.

The transformation stability metrics provide vital guidance for establishing registration extent limits. With affine RMSE-DEM values of 5–10 pixels for LightGlue, extrapolation beyond the reference coverage introduces positional uncertainties of 2.75–5.5 m at 0.55 m GSD. Operational workflows should constrain registration to regions with adequate reference coverage, segmenting large satellite scenes into tiles registered independently.

Beyond these per-metric observations, the experimental matrix produces a single engineering-level conclusion that is the practical deliverable of this study. The panchromatic-derived composites introduced here—SPECPAN (a spectral overlap-weighted PAN proxy) and EMPPAN (an RGB-only pseudo-PAN tuned for road surface contrast)—yield the highest verified-inlier density and the lowest cross-method variability of any of the seven evaluated band representations, while the affine RMSE-DEM metric reveals that LightGlue + CLAHE on these composites is also the most stable choice when the estimated transformation is extrapolated beyond the VWorld reference footprint. These two findings are not a per-method ranking but a decision rule for HD map preprocessing pipelines. Throughput-dominated, fully automated tiles should be processed with LightGlue + CLAHE on EMPPAN or BT601; precision-critical tiles where human review is acceptable should be processed with SIFT + CLAHE on EMPPAN; EdgeFFT should be avoided for cross-resolution satellite-to-aerial registration at patch sizes beyond ~350 px; and CLAHE should be applied selectively—as a hard precondition for LightGlue and as an optional enhancement for SIFT. This rule, summarized as a (band × method × patch size) decision table in the Supplementary Materials, is the form in which the present results are intended to be received by operational pipelines.

### 5.4. CLAHE Preprocessing Effects

The impact of CLAHE preprocessing varied substantially across methods. For SIFT, CLAHE consistently improved performance by enhancing local contrast and enabling feature detection in shadowed or low-contrast regions. The NIR band showed the greatest benefit, with inlier counts more than doubling after CLAHE application, confirming previous findings that histogram equalization supports SIFT keypoint detection under adverse illumination conditions [46].

Conversely, EdgeFFT methods exhibited degraded performance with CLAHE preprocessing. The histogram modification appears to disrupt the phase correlation process by altering the edge magnitude distribution. For frequency domain matching, original radiometric characteristics should be preserved, with alternative preprocessing approaches, such as edge enhancement, considered if contrast improvement is necessary.

LightGlue with CLAHE demonstrated robust performance, though direct comparison with non-CLAHE LightGlue was not included in this study. The deep learning-based feature detector may be inherently robust to contrast variations through learned invariances, potentially reducing the need for explicit preprocessing.

Beyond HD map preprocessing, robust geometric registration is also important for multi-temporal remote sensing analysis. In change detection pipelines, residual misregistration between image pairs can produce false changes and degrade accuracy, particularly near object boundaries, roads, and other high-contrast linear structures [47]. The same co-registration requirement applies to recent remote sensing image change captioning and vision language methods, which generate natural language descriptions from bi-temporal image pairs and, therefore, depend on geometrically consistent inputs [48,49]. The dense or detector-free learned matchers evaluated in this study, including LoFTR and RoMa, can provide spatially distributed correspondences for the alignment stage, while the observed effects of spectral representation and preprocessing offer practical guidance for registration front ends. Although downstream change detection or change captioning performance was not directly evaluated here, this connection situates the present satellite-to-aerial registration study within the broader remote sensing change analysis literature and motivates future work linking registration accuracy to semantic change analysis performance.

### 5.5. Limitations and Future Work

Several limitations of this study suggest directions for future research. As the evaluation focused on a single study area and a specific sensor pair (KOMPSAT-3A and VWorld), validation across diverse geographic regions and multiple satellite platforms is necessary to strengthen generalizability. Additionally, while the affine transformation model is computationally efficient, it may be insufficient for large scenes or areas with significant terrain variation. Investigation into higher-order transformation models or local adaptive methods warrants further study.

The experiments were conducted once for each spectral band, matching method, and patch size configuration using a deterministic processing pipeline. Therefore, the reported mean values summarize performance across evaluated experimental conditions, but they should not be interpreted as estimates from repeated independent trials. Confidence intervals and formal statistical significance tests were not applied in the current study because repeated experiments over independent scenes were not available. Future work should include repeated experiments across multiple scenes, regions, seasons, and sensor combinations to quantify variance and support statistical significance testing.

**Figure 4:**
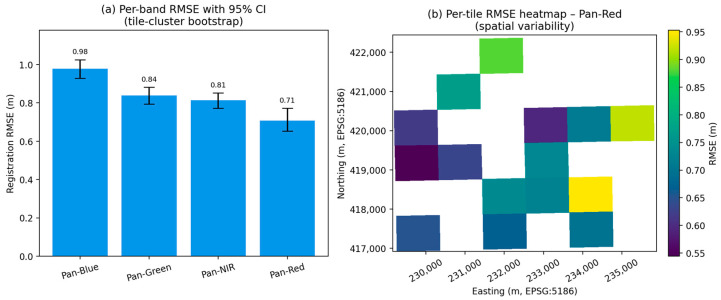
Statistical reliability of the spectral band comparison (representative LightGlue + CLAHE patch configuration). (**a**) Per-band registration RMSE with 95% confidence intervals from a tile cluster bootstrap (4000 replicates over a 6 × 6 tile grid). (**b**) Per-tile RMSE heatmap for the best-performing band (pan-red), showing spatial variability of about 0.5–1.0 m. The red band is significantly more accurate than the other bands (paired-tile bootstrap, 95% CI excluding zero).

Although each configuration was executed once, the spatial bootstrap provides confidence intervals and significance tests for the comparative conclusions (Figure 4). For the representative patch configuration, the per-band registration RMSE and 95% confidence intervals were 0.98 m [0.93, 1.03] for pan-blue, 0.84 m [0.79, 0.88] for pan-green, 0.81 m [0.77, 0.85] for pan-NIR, and 0.71 m [0.65, 0.77] for pan-red. The red band achieved the lowest RMSE, and the spatially paired bootstrap confirmed that it was significantly more accurate than pan-blue (+0.26 m [+0.18, +0.35]), pan-green (+0.11 m [+0.04, +0.17]), and pan-NIR (+0.12 m [+0.04, +0.19]); all confidence intervals excluded zero. The per-tile RMSE heatmap (Figure 4b) shows a spatial variability of approximately 0.5–1.0 m across the scene, with larger residuals toward the scene margins, consistent with the extrapolation behavior discussed above. These results indicate that the spectral band ranking is statistically robust rather than an artifact of a single run.

Although the affine transformation model is computationally efficient and useful for evaluating global registration behavior, it accounts only for global linear distortions and may not fully correct local nonlinear errors, terrain-related displacement, or spatially varying RPC bias. Therefore, the achieved accuracy should be interpreted as suitable for preprocessing and candidate GCP generation rather than final lane-level HD map production. To bridge the gap between the current 1.3–2.5-pixel registration accuracy and the 10–20 cm accuracy required for HD maps, future work should investigate higher-order polynomial transformations, Thin Plate Spline (TPS), local adaptive transformations, and rigorous RPC bias refinement. These methods should be evaluated using independent ground control and lane-level validation data.

**Figure 5:**
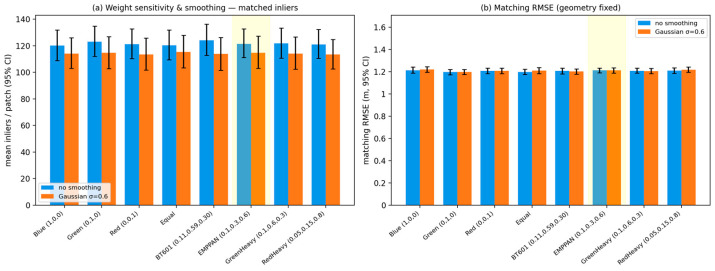
EMPPAN weight sensitivity and Gaussian-smoothing ablation. Co-located KOMPSAT-3A and VWorld patches at matched control point locations were matched with SuperPoint + LightGlue under eight RGB weightings, each with and without σ = 0.6 Gaussian low-pass filter applied uniformly (60 patches; error bars are 95% bootstrap confidence intervals). (**a**) Mean matched inliers per patch; (**b**) matching RMSE. EMPPAN (highlighted) performs comparably to the other weightings, and the smoothing slightly reduces inliers without improving RMSE.

The weight sensitivity and smoothing ablation (Figure 5) shows that the spectral weighting has only a minor influence on matching performance. Across all eight RGB weightings, the mean inlier count varied within a narrow 120–124 range (overlapping 95% confidence intervals), and the matching RMSE was essentially constant at 1.20–1.22 m. EMPPAN, therefore, performs comparably to the other visible band composites, and its heuristic weights are not a critical or finely tuned choice. Applying the σ = 0.6 Gaussian low-pass filter uniformly slightly reduced the inlier count (by roughly 6%) without improving the RMSE, indicating that the smoothing is not the source of EMPPAN’s correspondence density and is, if anything, marginally detrimental. These results confirm that the spectral band comparison is not confounded by the weighting or the smoothing operation. We note that this ablation isolates the radiometric effect under fixed geometry using the visible bands; the broader-spectrum SPECPAN composite, which additionally incorporates the NIR band, is evaluated separately in the main experimental matrix.

**Figure 6:**
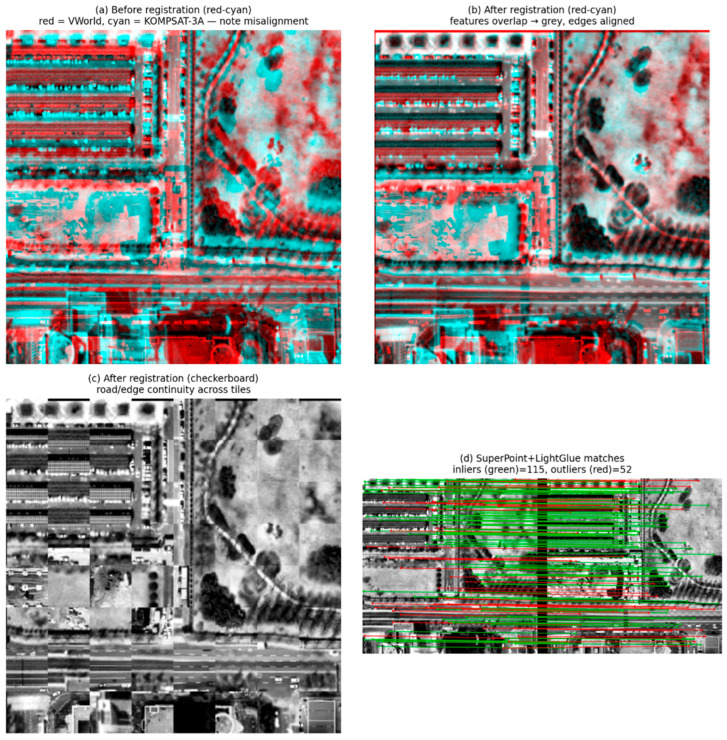
Qualitative registration assessment for a representative urban tile (≈200 m). (**a**) Red–cyan overlay before registration (red = VWorld, cyan = KOMPSAT-3A), showing the systematic offset; (**b**) red–cyan overlay after registration (red = VWorld, cyan = KOMPSAT-3A); (**c**) checkerboard mosaic after registration; (**d**) SuperPoint + LightGlue correspondences with RANSAC inliers (green) and outliers (red). Residual color in (**b**) reflects genuine temporal change between the non-simultaneous acquisitions.

To complement the quantitative metrics, Figure 6 provides a qualitative assessment of the registration for a representative urban tile (≈200 m). Before registration, the red–cyan overlay (Figure 6a) shows a clear systematic offset of about 8 m between the KOMPSAT-3A scene and the VWorld reference; after registration (Figure 6b), the two sources overlap into gray along building outlines and road edges, and the checkerboard mosaic (Figure 6c) shows continuous linear features across tile boundaries. The corresponding SuperPoint + LightGlue correspondences (Figure 6d) are dominated by geometrically consistent inliers (green) with a minority of rejected outliers (red). Residual colored areas in Figure 6b correspond to genuine temporal changes (vehicles, vegetation) between the non-simultaneous acquisitions rather than to misregistration.

**Figure 7:**
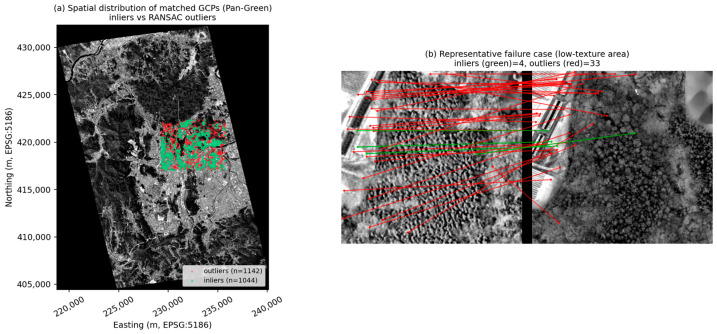
(**a**) Spatial distribution of matched GCPs (pan-green) over the KOMPSAT-3A scene: RANSAC inliers (green) and outliers (red), concentrated in the textured area covered by the VWorld reference. (**b**) Representative failure case in a low-texture vegetated area (four inliers, 33 outliers), where reliable correspondences cannot be established.

Figure 7a shows the spatial distribution of the matched GCPs for the pan-green band; inliers (green) and RANSAC-rejected outliers (red) are concentrated in the textured urban core covered by the VWorld reference, consistent with the spatial variability reported in Figure 4b. Figure 7b shows a representative failure case in a low-texture, vegetated area, where stable correspondences cannot be established (four inliers versus 33 outliers); such areas are correctly rejected by the RANSAC stage and would require alternative reference features or manual control points in an operational workflow.

**Figure 8:**
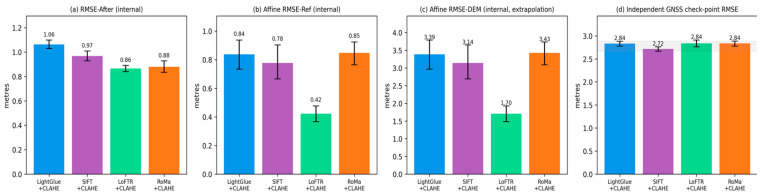
Feature-matcher comparison in the KOMPSAT-3A to VWorld pipeline (matrix-averaged over 7 spectral bands × 7 patch sizes; CLAHE preprocessing; identical sample locations; error bars are 95% bootstrap confidence intervals). (**a**) Matched-point RMSE After; (**b**) affine RMSE-Ref; (**c**) affine RMSE-DEM (extrapolation stability); (**d**) independent RMSE at the six network GNSS check points. LoFTR and RoMa improve the internal metrics ((**a**–**c**); LoFTR is the most stable), whereas the independent accuracy (**d**) is ≈2.8 m for all four matchers.

To broaden the deep learning comparison, two additional learning-based detector-free matchers were integrated into the identical registration pipeline: LoFTR (a semi-dense transformer matcher) and RoMa (a dense, DINOv2-based matcher representative of the current state of the art, superseding DKM). All four matchers were evaluated under matched conditions across the full 7 band × 7 patch size matrix with CLAHE preprocessing and identical sample locations (Table 8, Figure 8).

On the internal registration metrics, the detector-free matchers outperformed both LightGlue and SIFT. LoFTR and RoMa achieved the lowest matched-point RMSE After (1.57 and 1.60 px versus 1.93 px for LightGlue and 1.76 px for SIFT + CLAHE), and LoFTR provided markedly better transformation stability (affine RMSE-Ref 0.77 px and affine RMSE-DEM 3.09 px versus 5.7–6.2 px for the others; paired differences significant at 95% confidence). LoFTR and RoMa also yielded the highest correspondence density (≈2725 GCPs).

However, when evaluated against the six independent network GNSS check points (Section 3.1, Figure 3), all four matchers achieved statistically indistinguishable independent accuracy of approximately 2.8 m (Figure 8d): 2.84 m for LightGlue, 2.84 m for RoMa, 2.84 m for LoFTR, and 2.72 m for SIFT. The improvements in internal metrics, therefore, did not translate into better independent geodetic accuracy at the control points. The independent error is governed by the satellite RPC sensor model, the reference orthophoto accuracy, and the temporal and seasonal differences between acquisitions, rather than by the choice of matcher. Consistent with this, the independent check point RMSE correlated only weakly with the internal metrics among the well-registered configurations.

These results refine the practical guidance. The choice of feature matcher is primarily a throughput and robustness decision rather than an accuracy lever. LoFTR and RoMa are preferable where dense, stable correspondences are valuable—LoFTR additionally provides the most stable extrapolation—while LightGlue is attractive when computational efficiency is prioritized, and SIFT + CLAHE remains a competitive lightweight option; all four yield the same ≈2.8 m independent accuracy in the present KOMPSAT-3A/VWorld setting.

The temporal baseline between satellite and aerial acquisitions was not systematically varied in this experiment. The KOMPSAT-3A image was acquired on 28 March 2022, while the VWorld aerial orthophoto used as the reference dataset was from 2023. Although the reference orthophoto was selected because it was seasonally similar to the spring acquisition period of the satellite image, the datasets were not acquired simultaneously or under identical imaging conditions. Clouds, haze, weather conditions, acquisition time, solar position, seasonal effects, and temporal land cover changes are important factors affecting practical image registration performance. Although the selected test area did not show significant cloud contamination, the present results should be interpreted as performance under the available clear scene and seasonally similar reference conditions rather than as a complete assessment across all environmental conditions. These factors can affect local contrast, edge strength, texture consistency, and the number of reliable feature correspondences. Future work should extend the evaluation to multi-temporal, multi-seasonal, multi-regional, and multi-sensor datasets to assess transferability and operational robustness more objectively.

A primary limitation of this study is its restricted scope. All experiments were conducted on a single KOMPSAT-3A scene over Daejeon registered to a single VWorld aerial-orthophoto reference. The quantitative findings—optimal spectral bands, matcher behavior, patch size effects, and the approximately 2.8 m independent accuracy—should, therefore, be interpreted as specific to this KOMPSAT-3A/VWorld configuration rather than as universal operational rules. Land cover composition, seasonal and illumination conditions, terrain relief, and sensor-specific geometry are all expected to influence the results, and the decision guidance offered here should be revalidated before transfer to other regions or sensors. Cross-region, -season, and -sensor validation on the broader KARI KOMPSAT-3A archive and on additional VHR sensors (e.g., WorldView-3, Pléiades) is a necessary next step; accordingly, the operational claims have been tempered throughout the manuscript to reflect this single-setting evidence base.

## 6. Conclusions

This study presents a comprehensive evaluation of multi-spectral band performance for satellite-to-aerial image registration to support accurate spatial data generation for applications such as HD maps. Key findings include the following:

1. Spectral band selection significantly impacts registration performance. Panchromatic-derived products (EMPPAN, BT601) demonstrated superior stability and correspondence density compared to individual multi-spectral bands. Among visible bands, green and red achieved the optimal balance between feature matching and transformation accuracy. The two panchromatic-derived composites evaluated here—SPECPAN (spectral overlap-weighted) and EMPPAN (RGB-only pseudo-PAN)—were introduced in this work and showed the highest correspondence density and lowest cross-method variability across the full experimental matrix.
2. Among the matchers compared, the detector-free deep methods LoFTR and RoMa produced the densest and most stable correspondences, with LoFTR providing the best transformation stability (affine RMSE-DEM ≈ 3.1 pixels versus 5.7–6.2 pixels for the other matchers); LightGlue + CLAHE remained robust and computationally efficient, and SIFT + CLAHE offered competitive local accuracy (RMSE After ≈ 1.5 pixels). When validated against independent network GNSS check points, however, all matchers achieved a comparable registration accuracy of approximately 2.8 m, indicating that matcher selection is primarily a throughput and robustness decision rather than an accuracy decision.
3. Patch size configuration affects method-specific performance. LightGlue maintains stable accuracy across patch sizes, while SIFT benefits from larger patches that may compromise inlier rates. EdgeFFT methods are unsuitable for cross-resolution satellite-to-aerial registration due to degraded large-patch performance.
4. CLAHE preprocessing improves SIFT performance substantially, particularly for NIR bands, but actively degrades structural frequency domain (EdgeFFT) matching. Preprocessing must be algorithm-specific based on feature extraction characteristics.
5. Matched-point residuals of 1.3–2.5 pixels (0.7–1.4 m at 0.55 m GSD) were obtained, while an independent assessment against network GNSS check points indicated an absolute accuracy of approximately 2.8 m. Both confirm meter-level performance—roughly an order of magnitude coarser than the 10–20 cm lane-level HD map requirement—so additional RPC optimization, terrain correction, and downstream processing would be required for final HD map production, and the method is positioned for preprocessing and candidate GCP generation.

Rather than a ranked list of matchers, the deliverable of this study is an engineering decision rule of the form (spectral band × matching method × patch size), derived directly from the full 7 × 7 × 5 experimental matrix and from the within-footprint and extrapolation-stability metrics (affine RMSE-Ref and affine RMSE-DEM). The rule recommends LightGlue + CLAHE on EMPPAN or BT601 for throughput-dominated automated tiles, SIFT + CLAHE on EMPPAN for precision-critical tiles where human review is acceptable, avoidance of EdgeFFT at patch sizes beyond ~350 px, and selective application of CLAHE—mandatory for LightGlue, optional for SIFT. Cross-scene, cross-region, and cross-season validation of this rule on the broader KARI K3A archive and on additional VHR sensors (WorldView-3, Pleiades) is the immediate next step of this work. These recommendations are specific to the tested KOMPSAT-3A/VWorld configuration; the detector-free matchers LoFTR and RoMa are additionally preferable where dense, stable correspondences are valuable, with the caveat that all evaluated matchers yield a comparable independent accuracy of approximately 2.8 m.

## Figures and Tables

**Table 1:** Patch size configurations for experimental evaluation.

Config.	HRSI Patch (px)	VWorld Patch (px)	Ground Coverage (m)
1	131	289	72
2	169	373	93
3	201	433	110
4	351	773	193
5	427	939	235
6	501	1101	276
7	651	1433	358

**Table 2:** Feature-matching results for patch configuration 1 (HRSI: 131, VWorld: 289). The best value for each metric among the five methods is highlighted in bold.

Metric	Method	B	G	R	NIR	SPECPAN	BT601	EMPPAN
Total GCPs	LightGlue + CLAHE	**2001**	**2109**	**2175**	**2055**	**2158**	**2135**	**2160**
	EdgeFFT	735	834	884	884	875	858	866
	EdgeFFT + CLAHE	287	345	401	450	432	366	391
	SIFT	224	303	248	106	203	300	284
	SIFT + CLAHE	231	419	490	280	438	454	495
Inliers	LightGlue + CLAHE	**702**	**961**	**947**	**897**	**824**	**896**	**1046**
	EdgeFFT	248	223	184	344	310	297	275
	EdgeFFT + CLAHE	72	109	114	138	134	114	118
	SIFT	101	75	93	54	82	77	120
	SIFT + CLAHE	100	168	189	137	190	168	238
Inlier Rate (%)	LightGlue + CLAHE	35.1	**45.6**	**43.5**	43.6	38.2	**42.0**	**48.4**
	EdgeFFT	33.7	26.7	20.8	38.9	35.4	34.6	31.8
	EdgeFFT + CLAHE	25.1	31.6	28.4	30.7	31.0	31.1	30.2
	SIFT	**45.1**	24.8	37.5	**50.9**	40.4	25.7	42.3
	SIFT + CLAHE	43.3	40.1	38.6	48.9	**43.4**	37.0	48.1
RMSE After (px)	LightGlue + CLAHE	2.13	2.47	2.21	2.30	2.17	2.21	2.51
	EdgeFFT	2.30	1.83	1.68	2.17	2.02	1.98	1.93
	EdgeFFT + CLAHE	1.88	1.90	1.58	2.15	1.90	1.74	1.92
	SIFT	1.72	1.79	1.57	2.43	**1.50**	1.69	**1.67**
	SIFT + CLAHE	**1.64**	**1.60**	**1.29**	**1.68**	1.68	**1.49**	1.72
Improvement (%)	LightGlue + CLAHE	86.7	84.5	86.2	85.7	86.4	86.1	84.2
	EdgeFFT	86.9	89.5	90.2	87.3	88.2	88.6	88.7
	EdgeFFT + CLAHE	89.5	88.9	90.8	87.2	88.7	90.7	88.7
	SIFT	89.5	88.9	90.1	85.2	**90.8**	89.3	**89.4**
	SIFT + CLAHE	**89.9**	**90.2**	**91.9**	**89.5**	89.5	**90.8**	89.2

**Table 3:** Summary of registration performance by spectral band (averaged across methods). The best performance among the bands is highlighted in bold.

Band	Avg. Inliers	Avg. Rate (%)	RMSE After
Blue	288	39.3	1.95
Green	357	38.4	1.92
Red	353	34.6	**1.69**
NIR	368	**46.5**	2.15
SPECPAN	351	39.4	1.84
BT601	345	34.8	1.84
EMPPAN	**420**	42.6	1.96

**Table 4:** Feature-matching results for patch configuration 4 (HRSI: 351, VWorld: 773).

Metric	Method	B	G	R	NIR	SPECPAN	BT601	EMPPAN
Total GCPs	LightGlue + CLAHE	2758	2816	2845	2765	2820	2837	2849
	EdgeFFT	61	70	69	68	72	61	73
	EdgeFFT + CLAHE	58	78	60	71	73	73	52
	SIFT	632	968	907	306	663	979	982
	SIFT + CLAHE	862	1437	1634	939	1369	1564	1654
Inliers	LightGlue + CLAHE	1285	1408	1334	1347	1271	1131	1126
	EdgeFFT	33	61	57	52	60	36	65
	EdgeFFT + CLAHE	26	58	60	61	50	52	52
	SIFT	224	427	365	71	229	437	332
	SIFT + CLAHE	260	590	597	376	436	791	510
Inlier Rate (%)	LightGlue + CLAHE	46.6	50	46.9	48.7	45.1	39.9	39.5
	EdgeFFT	54.1	87.1	82.6	76.5	83.3	59	89
	EdgeFFT + CLAHE	44.8	74.4	100	85.9	68.5	71.2	100
	SIFT	35.4	44.1	40.2	23.2	34.5	44.6	33.8
	SIFT + CLAHE	30.2	41.1	36.5	40	31.8	50.6	30.8
RMSE After (px)	LightGlue + CLAHE	1.93	1.97	1.81	1.95	1.78	1.8	1.59
	EdgeFFT	12.99	16.62	16	15.11	16.87	11.63	18.08
	EdgeFFT + CLAHE	5.8	15.26	19.15	16.12	13.37	15.19	21.44
	SIFT	1.55	2.06	1.94	0.91	1.57	2.06	1.52
	SIFT + CLAHE	1.28	1.7	1.59	1.66	1.43	2.1	1.23
Improvement (%)	LightGlue + CLAHE	87.7	87.4	88.3	87.5	88.5	88.5	89.8
	EdgeFFT	28	12.2	17.5	18.1	12.4	40.1	7.1
	EdgeFFT + CLAHE	66.9	20.6	1.5	14.4	33.7	23.3	1.7
	SIFT	90.5	87.3	87.9	94.6	90.3	87.2	90.5
	SIFT + CLAHE	92.1	89.2	89.9	89.8	91	86.8	92.2

**Table 5:** Feature-matching results for patch configuration 7 (HRSI: 651, VWorld: 1433).

Metric	Method	B	G	R	NIR	SPECPAN	BT601	EMPPAN
Total GCPs	LightGlue + CLAHE	2847	2926	2951	2841	2938	2939	2929
	EdgeFFT	1	3	4	1	4	3	3
	EdgeFFT + CLAHE	23	24	29	25	37	29	24
	SIFT	984	1541	1546	525	1124	1625	1644
	SIFT + CLAHE	1366	2186	2486	1494	2058	2314	2444
Inliers	LightGlue + CLAHE	996	1130	1236	1058	1218	970	1256
	EdgeFFT	N/A	N/A	N/A	N/A	N/A	N/A	N/A
	EdgeFFT + CLAHE	N/A	16	14	17	21	10	19
	SIFT	321	607	498	197	522	708	694
	SIFT + CLAHE	442	1044	1065	508	735	890	953
Inlier Rate (%)	LightGlue + CLAHE	35	38.6	41.9	37.2	41.5	33	42.9
	EdgeFFT	N/A	N/A	N/A	N/A	N/A	N/A	N/A
	EdgeFFT + CLAHE	N/A	66.7	48.3	68	56.8	34.5	79.2
	SIFT	32.6	39.4	32.2	37.5	46.4	43.6	42.2
	SIFT + CLAHE	32.4	47.8	42.8	34	35.7	38.5	39
RMSE After (px)	LightGlue + CLAHE	1.58	1.55	1.85	1.58	1.74	1.31	1.95
	EdgeFFT	N/A	N/A	N/A	N/A	N/A	N/A	N/A
	EdgeFFT + CLAHE	FAIL	2.29	1.34	2.19	2.12	0.85	2.49
	SIFT	1.6	1.86	1.7	1.62	2.02	2.03	2.03
	SIFT + CLAHE	1.54	1.96	1.79	1.45	1.44	1.68	1.71
Improvement (%)	LightGlue + CLAHE	90.2	90.2	88.2	90	89	91.7	87.6
	EdgeFFT	N/A	N/A	N/A	N/A	N/A	N/A	N/A
	EdgeFFT + CLAHE	N/A	87	92.1	87.5	87.9	95.2	85.4
	SIFT	90.3	88.6	89.5	90.6	87.7	87.5	87.5
	SIFT + CLAHE	90.4	87.7	88.7	91	90.8	89.4	89.1

**Table 6:** Registration performance across patch size configurations (LightGlue with CLAHE, all bands averaged). The best performance among the configurations is highlighted in bold.

Config.	Avg. Inliers	Inlier Rate (%)	RMSE After (px)	Improvement (%)
1	896	42.3	2.29	85.7
2	1072	45.1	2.04	87.4
3	1233	**49.0**	2.23	85.9
4	1272	45.2	1.83	88.2
5	**1385**	48.1	1.99	87.3
6	1141	39.6	**1.54**	**89.6**
7	1123	38.6	1.65	**89.6**

**Table 7:** Transformation stability metrics (patch configuration 1).

Method	Metric	Min	Max	Mean	Std Dev	Relative Stability
LightGlue + CLAHE	RMSE-Ref	0.30	1.76	0.94	0.46	High
	RMSE-DEM	1.21	7.10	4.09	1.89	High
SIFT + CLAHE	RMSE-Ref	0.16	1.77	1.01	0.52	High
	RMSE-DEM	0.66	7.18	4.35	2.19	High
EdgeFFT	RMSE-Ref	0.18	2.47	1.16	0.82	Medium
	RMSE-DEM	0.72	9.98	4.40	3.21	Low
EdgeFFT + CLAHE	RMSE-Ref	0.38	4.04	1.83	1.36	Low
	RMSE-DEM	1.53	16.33	7.60	5.44	Low

**Table 8:** Detector-free deep matchers (LoFTR, RoMa) versus LightGlue + CLAHE and SIFT + CLAHE, matrix-averaged over the 7 × 7 band × patch matrix. Registration metrics in pixels (0.55 m GSD); independent check point (CP) RMSE in meters.

Matcher	Total GCPs	Inliers	RMSE After (px)	Affine RMSE-Ref (px)	Affine RMSE-DEM (px)	Indep. CP RMSE (m)
LightGlue + CLAHE	2655	1166	1.93	1.52	6.16	2.84
SIFT + CLAHE	1384	515	1.76	1.41	5.71	2.72
LoFTR + CLAHE	2725	889	1.57	0.77	3.09	2.84
RoMa + CLAHE	2512	1114	1.60	1.54	6.23	2.84

## Data Availability

All results presented in this study are available upon request from the corresponding author.

## Supplementary Materials

The following supporting information can be downloaded at https://www.mdpi.com/article/10.3390/s26134165/s1: Complete results for all seven patch size configurations. Failed or unavailable cases are marked as N/A or FAIL.

## Abbreviations

HD map: high-definition map; HRSI: high-resolution satellite imagery; GSD: ground sampling distance; RPCs: rational polynomial coefficients; GCP: ground control point; DEM: digital elevation model; CLAHE: contrast-limited adaptive histogram equalization; SIFT: scale-invariant feature transform; FFT: fast Fourier transform; NIR: near-infrared; SPECPAN: spectral overlap panchromatic simulation; BT601: ITU-R BT.601 luminance composite; EMPPAN: empirical RGB pseudo-panchromatic composite.
